# Exploiting Genetic Interference for Antiviral Therapy

**DOI:** 10.1371/journal.pgen.1005986

**Published:** 2016-05-05

**Authors:** Elizabeth J. Tanner, Karla A. Kirkegaard, Leor S. Weinberger

**Affiliations:** 1 Gladstone Institutes (Virology and Immunology), San Francisco, California, United States of America; 2 Department of Genetics, Stanford University School of Medicine, Stanford, California, United States of America; 3 Department of Biochemistry and Biophysics, University of California, San Francisco, San Francisco, California, United States of America; Baylor College of Medicine, UNITED STATES

## Abstract

Rapidly evolving viruses are a major threat to human health. Such viruses are often highly pathogenic (e.g., influenza virus, HIV, Ebola virus) and routinely circumvent therapeutic intervention through mutational escape. Error-prone genome replication generates heterogeneous viral populations that rapidly adapt to new selection pressures, leading to resistance that emerges with treatment. However, population heterogeneity bears a cost: when multiple viral variants replicate within a cell, they can potentially interfere with each other, lowering viral fitness. This genetic interference can be exploited for antiviral strategies, either by taking advantage of a virus’s inherent genetic diversity or through generating de novo interference by engineering a competing genome. Here, we discuss two such antiviral strategies, dominant drug targeting and therapeutic interfering particles. Both strategies harness the power of genetic interference to surmount two particularly vexing obstacles—the evolution of drug resistance and targeting therapy to high-risk populations—both of which impede treatment in resource-poor settings.

## Introduction

RNA viruses, such as influenza virus, HIV, and dengue virus, are dynamic, rapidly evolving pathogens that evade most therapeutic interventions. The error-prone viral RNA polymerase generates genetically heterogeneous viral populations that, in combination with large population sizes, enable these viruses to overcome static (chemical) drug therapies. Drug-resistant viral infections often emerge within days after treatment starts, the natural result of a strong selection pressure (the drug) acting on a genetically heterogeneous viral population [1–5]. Since RNA viruses are responsible for many of the world’s ongoing and emerging epidemics (e.g., HIV, dengue virus, Zika virus), strategies that withstand viral dynamics and evolution are required. Below, we discuss two such strategies that aim to therapeutically target viruses not by blocking viral enzymes or receptors but by interfering with the fundamental viral structure or assembly processes. These two interventions—based upon the relatively arcane viral phenomena of “phenotypic masking” and defective interfering particles—have the potential to greatly diminish, and possibly overcome, problems associated with drug resistance and access to therapy.

### Target for interference: virion capsid assembly

**Fig 1:**
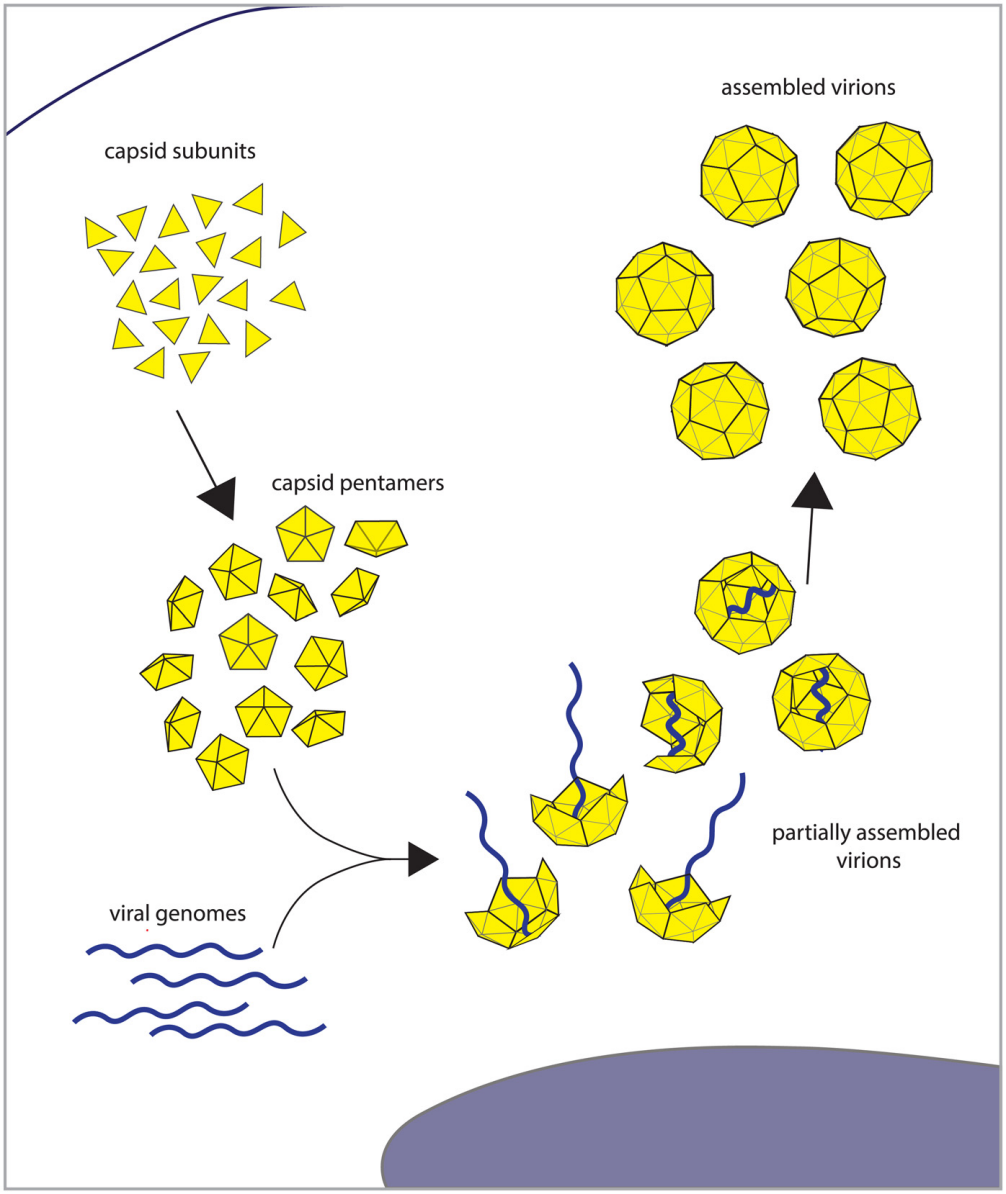
Schematic of targets of genetic interference: capsid assembly and genome encapsidation. Individual capsid subunits (yellow triangles) assemble into pentamers, which assemble to form the full icosahedral capsid. The viral genome (blue) is packaged into the capsid.

All virions are assembled from a few essential components: the viral genome, a protective protein shell called the capsid, and often an outer lipid envelope. Capsids are self-assembling oligomers composed of hundreds to thousands of individual proteins (i.e., they are homo-oligomers) (Fig 1). Assembled capsids conform to a variety of shapes. For example, many RNA virus capsids are icosahedral, while the HIV capsid is conical, and the flaviviruses, such as dengue virus, have a disordered capsid often referred to as the core [6–10]. Regardless of their topology, capsids are high-order oligomers composed of identical subunits that self-assemble with high apparent cooperativity [11].

### Capsid heterogeneity and “phenotypic masking”

The homo-oligomeric nature of capsid formation makes capsid assemblies a rich target for genetic interference. Capsid assembly proceeds from a common pool of proteins, and mature capsids are often chimeras assembled from the proteins of multiple viral variants within a cell. In some cases, one variant may be susceptible to interference by toxic capsid subunits generated by another viral variant (i.e., dominant-negative interactions). For example, when antibody-resistant variants replicate in cells alongside antibody-sensitive variants, chimeric capsids can form. The antibody-sensitive capsid subunits in the structure can be bound by the antibody, leading to neutralization of the entire virion, despite the presence of escape-variant capsid subunits within the virion [12].

This dominant-negative interaction between antibody-sensitive and antibody-resistant capsid subunits is referred to as phenotypic masking. As described below, the formation of chimeric capsids can be exploited as a therapeutic strategy to suppress drug-resistant variants.

### Target for interference: encapsidation of viral genetic material

In most cases, the viral genome is packaged through direct interactions with viral packaging proteins, such as the capsid. For some RNA viruses, much of the specificity of packaging likely derives from direct coupling of packaging to RNA synthesis; only newly synthesized genomic RNAs are packaged. In many other cases, the packaging proteins recognize specific nucleotide sequences embedded in the viral RNA, regardless of the age or origin of the RNA. For example, the HIV-1 genome encodes *cis*-acting packaging signals, including the psi packaging element [13–16], reviewed in [17]. In contrast to the packaging signal, many viral proteins, including capsid and envelope proteins, act in *trans*. Hence, any RNA that encodes the requisite *cis*-packaging sequences can be packaged into the HIV-1 capsid, including reporter constructs, deletion mutants, and even RNAs of unrelated viruses [13]. This ability to encapsidate orthogonal genetic elements into a given viral capsid enables viral vectors to be used for gene delivery and gene therapy but also provides an opportunity for genetic interference.

### Parasitic genome encapsidation by interfering particles

Genetic interference via encapsidation is feasible because virion assembly often entails mass-action processes [11,18,19] that proceed regardless of the source of the raw materials provided. In lentiviruses, for example, orthogonal capsid proteins, envelope proteins, and even genomes can be “spiked” into a cell from various sources, processed, and integrated into virion assembly in essentially the same manner as raw materials from any other source (standard complementation assays between viral mutants also suggest that viral variants within a cell share a common pool of viral products). Of course, not all viral processes necessarily proceed from a well-mixed pool of viral proteins (e.g., certain poliovirus proteins required for genome synthesis are not efficiently complemented in *trans* [20]). Nevertheless, in viral processes where mass-action mixing readily occurs, if two viral variants are present in a cell, their genomes inevitably compete for shared viral products, such as capsid and envelope proteins [21,22].

This competition between viral genomes can generate a fitness cost, as illustrated by a phenomenon described in the 1940s by both Henle and von Magnus. During serial passaging of influenza virus at high multiplicities of infection (MOI), they observed the formation of “incomplete” viruses that decreased the overall yield of infectious virus [23,24]. In 1970, the term defective interfering particle (DIP) was coined to describe these incomplete genomes, which are replication-incompetent and depend on a complete helper virus for replication. For example, influenza DIPs are typically composed of genomes in which one or more segments contain internal deletions [25], often in the polymerase genes [26], and thus rely on a helper virus to provide the missing polymerase.

Many other single-stranded RNA viruses, including dengue virus, poliovirus, and vesicular stomatitis virus, were subsequently found to produce DIPs [27–29]. However, it took decades to determine the mechanism of interference: deletion variants acting as molecular parasites of the wild-type virus [30]. Importantly, DIP genomes tend to be more abundant in the cell because of a replication advantage provided by their smaller size or increased affinity for the polymerase [31–35]. Since genome encapsidation is typically a mass-action process, the more abundant DIP genomes tend to stoichiometrically outcompete the wild-type genomes for packaging proteins [30,36–38]. Overall, these cheater variants (i.e., DIPs) amplify themselves at the expense of the wild-type virus [39,40]. This phenomenon is exploited for the therapeutic interfering particle (TIP) strategy discussed below.

## Harnessing Genetic Interference for Antiviral Therapy

### Exploiting phenotypic masking for dominant drug targeting: experimental validation

Since the replication processes of RNA viruses are highly error-prone, genetic variants are unavoidable and rapidly arise. These variants generate resistance to virtually any monotherapy. However, the existence of drug-resistant variants does not guarantee their selection, and genetic interference can ensure that these mutants are not selected. For example, as explained below, targeting capsid or self-assembly processes with an inhibitor enables wild-type, drug-susceptible genomes to naturally suppress the outgrowth of drug-resistant variants [41,42]. The term dominant drug target was coined to highlight the dominant-negative role of the drug-susceptible variant.

**Fig 2:**
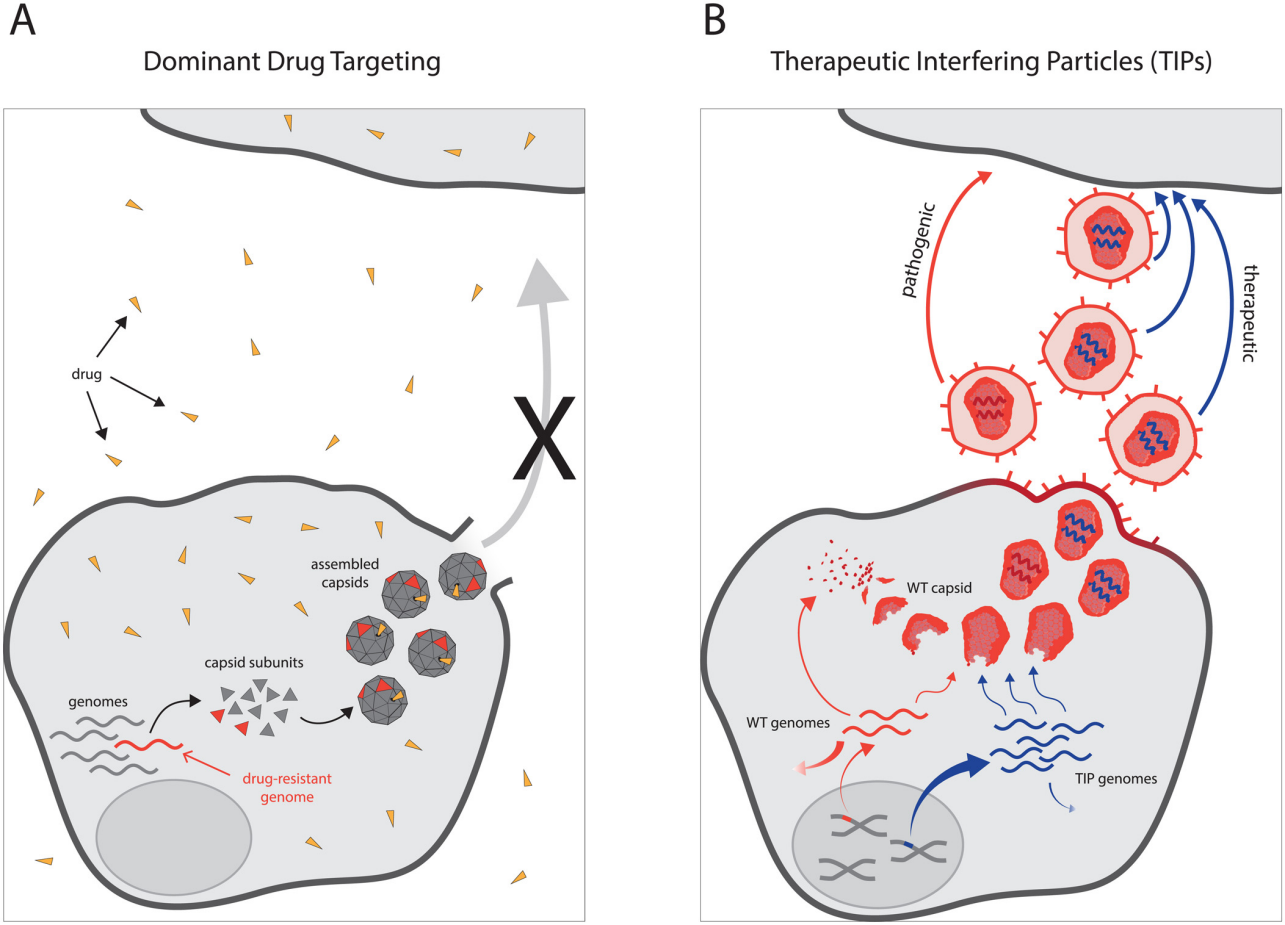
Exploiting genetic interference in capsid assembly and encapsidation for antiviral therapy. (A) Dominant drug targeting. Drug-resistant genomes (red curves) arise from wild-type genomes (gray curves) during replication within an infected cell. Drug-resistant and drug-sensitive genomes generate capsid proteins (red and gray triangles, respectively), which assemble into chimeric capsids. The drug (orange triangle) interferes with the function of both wild-type and chimeric capsids. (B) Therapeutic interfering particles (TIPs). After reverse transcription and integration, the HIV-1 proviral genome (red line) is integrated within the host genome and generates cytoplasmic mRNAs (red curves) by using its *trans* elements Tat and Rev as well as host cofactors. These mRNAs translate viral proteins, including capsid (red), and some of the mRNAs also serve as genomic RNA and are encapsidated in diploid form. TIP genomes (blue line) carry large deletions but are also reverse transcribed and integrated into the host genome. TIPs also utilize wild-type HIV-1 *trans* elements (e.g., Tat and Rev) to express genomic RNAs in the cytoplasm (blue curves). Since they have extensive deletions, these RNAs encode few if any proteins but are transcribed in higher proportions than wild-type RNAs. Because they are present at higher concentrations, TIP RNAs can outcompete wild-type virus (red) for packaging proteins and encapsidate their own diploid genomes in place of HIV-1 genomes.

Dominant drug targeting is based on the fact that when a drug-resistant variant first arises within a cell (through the error-prone replication of the viral genome), it is most often a minority variant in a population of wild-type, drug-susceptible genomes generated during genome amplification in that cell (Fig 2A). When an oligomeric structure such as the capsid is the drug target, both drug-susceptible and drug-resistant genomes contribute products to the common pool of proteins, leading to the assembly of capsids that contain both drug-resistant and drug-susceptible subunits. Thus, chimeric capsids—susceptible to drug inhibition despite the presence of resistant subunits—are likely to form in each cell in which drug-resistant genomes arise, and the outgrowth of the drug-resistant mutants is likely halted.

The concept of dominant drug targeting has been demonstrated for both poliovirus and dengue virus using capsid and core inhibitors, respectively [41,42]. Targeting these oligomeric structures with inhibitors suppressed the emergence of drug resistance in infected mice. In striking contrast, targeting proteins involved in genome replication resulted in the emergence of drug resistance within days of initiating treatment. The efficacy of the capsid and core inhibitors could not be attributed to a lack of resistance mutations; resistant viral variants arise frequently during replication, and a single nucleotide change confers full resistance to either drug [41,43–45]. For both of these dominant drug targets (capsid and core), biochemical analyses confirmed the formation of oligomeric complexes susceptible to drug inhibition (e.g., Tanner et al. [41] directly show formation of chimeric, drug-inhibitable poliovirus capsids). These studies were the first to show that dominant drug targeting can suppress the emergence of drug resistance.

### Expanding on dominant drug targets

Given that the dominant drug target strategy has been validated for two different virus families (one enveloped and one nonenveloped), the strategy is likely to be broadly applicable to other viruses and targets. Two key conditions must be met for dominant drug targeting to be successful: (i) the virus must exhibit intracellular heterogeneity, and (ii) the target must be susceptible to dominant-negative interactions. Condition (i) is met by most single-stranded RNA viruses. These viruses rely on error-prone polymerases that introduce new mutations into each progeny genome, thereby generating genetic heterogeneity within an individual infected cell. Condition (ii) can be empirically tested by screening for viral factors susceptible to dominant-negative interactions.

A screen in poliovirus identified viral factors other than the capsid that are susceptible to dominant-negative interactions [46]. These targets included elements involved in genome replication, such as the viral polymerase and an RNA structure that acts as a template for genome synthesis. Curiously, only some polymerase mutants were able to act as dominant negatives, suggesting that only some functions of the polymerase are susceptible to genetic interference. While it remains unclear how genome synthesis can be susceptible to dominant-negative interactions, one clue may lie in the fact that, in many viruses, the polymerase forms an oligomer [47–50]. These oligomers are functional, and RNA replication is cooperative, suggesting that multiple polymerase molecules may replicate the same template simultaneously [51–54]. Perhaps defective subunits within the polymerase oligomer act as toxic subunits. Similarly, an intramolecularly cleaving protease identified in the screen may generate a toxic fusion protein by failing to cleave itself from the viral polyprotein precursor. Given that the poliovirus screen identified multiple factors susceptible to dominant-negative interference, similar screens in other viruses may illuminate additional dominant drug targets.

Moving forward, targeting inhibitors to dominant drug targets could reduce the number of antivirals required to treat infections and substantially lower the chance of resistance for the targeted viral pathogen. These factors could lower the cost and potential side effects of antiviral therapy regimens.

### Potential pitfalls of dominant drug targeting

Dominant drug targeting relies on inherent viral interactions that must be sufficiently strong to suppress drug resistance over the course of treatment. If between-variant interactions are rare (perhaps because of insufficient mixing of proteins), the drug-resistant genome may escape suppression. Any drug-resistant variant that seeds a new round of infection in the absence of drug-susceptible genomes will no longer be controlled by genetic interference. Given that drug resistance did not emerge from the treated mice [41,42], mixing appears to be common in the capsid and core of poliovirus and dengue virus, respectively, and interference within these processes is quite effective. Nevertheless, these mouse studies were carried out on the order of days, so the long-term efficacy of dominant drug targeting has not been assessed. Modeling the probability that an infection escapes suppression could help determine how susceptible each target will be to drug failure and the potential consequences for population-level infection control.

## Generating De Novo Interference for Therapy

### Theory: therapeutic interfering particles

Overcoming drug resistance remains a critical challenge. However, for many communicable diseases, two other challenges are at least as problematic: behavioral barriers, such as poor adherence to therapy and risk disinhibition in the target populations, and unequal deployment of therapies, which results from differences in access to therapy in at-risk populations. These three challenges—resistance, adherence, and access—arise from three core characteristics inherent to viruses but absent in antiviral interventions: evolution (viruses mutate, but pharmaceuticals do not, invariably leading to resistant variants), persistence (viruses evolved mechanisms to remain in the host, but pharmaceuticals are cleared, necessitating sustained adherence), and transmission (viruses are communicable, but pharmaceuticals are not, so deployment of therapies always lags).

These challenges are exemplified by the HIV-1/AIDS pandemic. HIV-1 rapidly evolves resistance [55,56], often necessitating cocktails of three antiviral drugs to effectively suppress viral replication. HIV-1 also establishes lifelong persistence in infected persons, typically requiring lifelong adherence to therapeutic regimens [57]. Finally, HIV-1 transmission patterns are highly heterogeneous, with hard-to-reach superspreaders (e.g., commercial sex workers and their clients) who engage in high-risk behaviors, contributing disproportionately to disease spread [58–60]. Hence, despite the development of highly suppressive antiretroviral drugs, effective pandemic control has been unattainable, and some 2 million people are infected each year, primarily in resource-limited settings, such as sub-Saharan Africa [61].

A proposed solution to overcome these challenges is to utilize the concept of DIPs by engineering optimized TIPs [62] that molecularly parasitize wild-type viruses across multiple biological scales, from the cellular to the epidemiological scale. While proposals to use DIPs for therapy are not new [63–68], the hypothesis that DIPs engineered to stoichiometrically outcompete wild-type viruses at the cellular scale could lessen disease at the epidemiological scale [62] is a unique therapeutic proposition.

Provided that TIPs can be engineered, theoretical models predict that for HIV/AIDS, TIPs could piggyback on wild-type HIV-1 replication (Fig 2B) to produce substantially greater reductions in disease prevalence compared to conventional interventions. Specifically, these data-driven models linked molecular, patient, and epidemiological scales and assessed the feasibility of TIPs as population-level HIV-1 therapeutic interventions compared to conventional vaccines and drug therapies [62,69–71]. Strikingly, the epidemiological analyses predicted that introducing TIPs into a small percentage of infected individuals could significantly lower the HIV/AIDS prevalence, particularly because TIPs would automatically concentrate in the superspreader populations that are so challenging to reach with conventional therapies and vaccines [62].

The fundamental reason that TIPs outperformed conventional interventions in silico was based on the ability of DIPs to transmit through human populations [72]. Consequently, TIPs could disseminate through natural HIV transmission networks, despite the model accounting for a lower transmission efficacy of TIPs than HIV-1. Being lentiviral vectors, TIPs integrate into the host genome and can lie latent [73,74] for the lifetime of the cell until subsequent wild-type virus infection. Hence, despite DIP transmission being less successful when viral loads are low (i.e., during initial infection), TIPs have the potential to reactivate upon subsequent wild-type virus infection. Accounting for this long-lived latent state increases effective TIP transmission and reduces HIV/AIDS prevalence in epidemiological models [62], but in high-risk populations, TIPs retain efficacy even absent latency [62,71].

Remarkably, the models also showed that HIV-1 is unlikely to escape TIP parasitism through adaptation because the two populations experience competing selection pressures. For example, HIV-1 could theoretically escape TIP interactions by altering the specificity of packaging. Such a functional change would require at least two independent mutations, one in the packaging signal and one in the capsid. However, TIPs are under pressure to maintain their interaction with HIV-1 packaging proteins and will only have to make the single mutation in the packaging signal to bind an altered capsid protein. Being dependent on the same error-prone replication machinery as HIV-1, TIPs would adapt at the same evolutionary rate as HIV-1 and would be able to maintain the parasitic interaction. Extensive modeling of various escape scenarios suggests that HIV-1 cannot escape the TIP interaction without severely limiting its own growth [69–71]. Hence, TIPs may constitute “resistance-proof” antivirals.

### Constructing TIPs for HIV-1 and beyond

While a bona-fide TIP has not yet been experimentally validated, a number of HIV-based vectors have been engineered to interfere with viral replication [75–80]. These engineered vectors partially fulfill the criteria of a TIP.

The models [62,69–71] indicate that, for therapeutic effectiveness and halting resistance, TIPs must exhibit a basic reproductive number (R_0_) > 1 (specifically, a within-patient R_0_ > 1). This criterion means that TIP-infected cells must, on average, stably and continually generate more than one new TIP-infected cell. Theoretically, TIPs that have an R_0_ > 1 necessarily reduce the HIV-1 R_0_, thereby reducing HIV-1 viremia in patients and ultimately reducing HIV-1 transmission and prevalence.

Molecularly, the design criteria that enable R_0_ > 1 are: (i) that excess TIP genomic RNA (gRNA) be generated compared to wild-type HIV-1 gRNA within an HIV-1 infected cell and (ii) that the excess TIP gRNAs encode all relevant *cis* elements required for efficient replication and packaging. These two design criteria ensure that the TIP genome will spread from one cell to the next more efficiently than its helper (HIV-1) virus. The excess (or asymmetry) of TIP gRNA synthesis can be achieved by enhanced production or diminished degradation of TIP gRNA, compared to wild-type HIV-1 gRNA (Fig 2B). For HIV, abrogating mRNA splicing can cause more RNA to remain as gRNA [81] and could thus satisfy the requirement for TIP gRNA production asymmetry. The resulting excess of TIP gRNA would enable TIPs to outcompete HIV-1 for encapsidation resources in infected cells.

Unfortunately, the previously constructed therapeutic vectors [75–80] encode genetic elements that directly antagonize HIV replication, likely precluding R_0_ > 1 because production of essential *trans* elements is inhibited [82]. For example, the Dropulic group designed an HIV-based vector that expresses antisense RNA to the virus envelope transcript [83,84]. This vector mobilizes in patients to some extent, but viral suppression was temporary, and the vector was lost over time [85,86]. Nevertheless, the fact that these vectors transiently mobilized in patients provides a rationale that TIPs may be engineered against HIV-1. Moreover, HIV-1 deletion mutants lacking HIV-inhibitory elements also inhibit the spread of HIV-1 in cell culture and mobilize in the presence of a wild-type virus [76,78], but these mutants were selected based on inhibition (not transmission), and so it is unclear if R_0_ > 1 was satisfied.

Beyond HIV-1, interventions that address the universal barriers to disease control (i.e., resistance, adherence, and access) will likely be critical to control future epidemics of other viruses (e.g., retro- or lentiviruses that zoonotically cross over from nonhuman primates). Retroviruses related to HIV-1 are endemic to primate species in Africa, and 12 cases of retroviral zoonotic transmissions from nonhuman primates to humans have been documented in the last century [87–92]. Given that one of these transmissions is responsible for the HIV-1 pandemic, concern over future retroviral pandemics is not unfounded; another pandemic virus may emerge from the primate reservoir and overwhelm conventional disease-control approaches, as did HIV-1. Hence, development of TIP-like interventions capable of overcoming the barriers to effective disease control would seem prudent.

### Potential pitfalls of the TIP strategy

The safety and ethical implications of transmissible, integrating therapies are important considerations that have been addressed extensively by Notton et al., [93], but we briefly summarize the main points here.

The chief concerns center around: (i) safety risks associated with insertional mutagenesis of TIPs into the host genome and potential oncogenic transformation of the cell, (ii) viral evolution and recombination, leading to more virulent HIV-1 strains, and (iii) the ethical implications of treating individuals without consent.

While insertional mutagenesis leading to oncogenesis was problematic with early gamma retroviral gene therapy in hematopoietic stem cells [94], this genotoxicity appears limited to the specific combination of gamma retrovirus and hematopoietic stem cells. Carl June and colleagues found no evidence of oncogenesis or clonal dominance in a decade-long trial in human patients [95] when retroviral vectors were used on terminally differentiated T cells. For lentiviral gene therapies, no examples of oncogenesis have been reported when either differentiated or undifferentiated cell types have been transduced in the clinic [85,86,95–98]. Multiyear follow-up studies of patients transduced with lentiviral vectors targeting hematopoietic stem cells—highly transformable cell types—showed no genotoxicity or preferential expansion of transduced cells in patients [97,98]. The genotoxicity of the original gamma retroviral vectors in hematopoietic cells may arise from their differential promoter architecture compared to lentiviruses and the proclivity of retroviruses to integrate near the 5′ transcription start sites as well as in proto-oncogenes, while the lentiviral patient trials noted no increased integration events near the 5′ ends of genes or near proto-oncogenes (the integration patterns were quasi-random in genic regions). Given that TIPs are lentiviruses and would infect the same cells as HIV-1 (primarily differentiated T cells), the patient trials above suggest that potential therapeutic benefits of TIPs may outweigh risks associated with insertional oncogenesis.

The risk that TIPs may lead to the evolution of increased virulence in HIV-1 is a theoretical possibility that has been investigated using quantitative evolutionary dynamics models [62,69–71]. These models indicate that it is unlikely that HIV-1 could evolve away from TIPs, at either the patient [69] or epidemiological scale [71]. Intuitively, the modeling results can be understood from the fact that TIPs parasitize and replicate more efficiently than HIV-1; while HIV-1 needs to mutate both *cis* and *trans* factors simultaneously to escape TIPs, TIPs only mutate *cis* factors (they lack *trans* factors). This two-to-one mutation rate advantage largely precludes HIV-1 escape variants at the individual-patient scale. Moreover, the models argue that recombination with wild-type HIV, while certainly possible, is selected against by both HIV-1 and TIPs [69]. In the event that recombination occurs, it will be limited to already infected individuals, and even if it occurs in every infected cell, it will ultimately reconstitute the already present wild-type virus (i.e., the equivalent of an HIV mutation that results in loss of efficacy for a therapy).

While the notion of a transmissible therapy—even a therapy such as TIPs that only transmit in a limited fashion between already infected individuals—may raise ethical concerns regarding consent, many of these ethical issues have already been addressed for live-attenuated vaccines. For example, the oral poliovirus vaccine (OPV) is a live-attenuated vaccine chosen by the World Health Organization (WHO) for the worldwide polio eradication effort [99] (most recently, OPV successfully eradicated polio in India [100]). Like many live-attenuated vaccines, OPV is known to transmit between individuals—vaccinated individuals shed OPV virus for days, inoculating other individuals against the poliovirus—and this transmission of vaccination is widely viewed as a benefit of OPV. While there are safety risks associated with live-attenuated vaccines such as OPV (i.e., reversion to virulence), TIPs exhibit far greater safety in this regard, as they can only recombine to become virulent if they have mobilized (i.e., in individuals already infected with wild-type virus). Hence, for TIPs, the consent and reversion issues are circumscribed to the infected populations as opposed to the general populace.

All medical interventions carry inherent risk. Interventions that enter into clinical practice are those in which the benefits have been determined to outweigh the risks. Risk–benefit analysis is thus an essential aspect of any medical innovation. Overall, while the safety risks and ethical issues associated with TIPs cannot be overlooked, given the potential benefits TIPs may provide in overcoming universal disease control barriers and the precedents above, not pursuing research on these potential therapeutic interventions may ultimately be more ethically problematic.

## Conclusion

The dominant drug targeting and TIPs strategies employ different mechanisms, but both rely on genetic interference to counter viral population heterogeneity, lower the viral burden, and overcome the obstacles that face current antiviral therapies. Dominant drug targeting operates by clearing an infection with chemical inhibitors targeting highly cooperative processes, and TIPs control infection at multiple biological scales (molecular to epidemiological) through a transmissible molecular parasite particle. The dominant drug targeting strategy exploits naturally occurring dominant-negative interaction to suppress the key target—drug-resistant genomes. Such dominant-negative interactions require intracellular heterogeneity. The TIP strategy, on the other hand, introduces a molecular parasite into the population and harnesses genetic interference through parasitic genome encapsidation, so TIPs can target viruses that generate intercellular genetic diversity even if these viruses do not exhibit intracellular diversity. These therapeutic strategies based on genetic interference have the potential to overcome major barriers in disease control, including drug resistance and population-level barriers to effective deployment of therapy. Going forward, quantitative viral genetics approaches may expose new targets and strategies to exploit genetic interference for therapy.
